# Supplementary material for: COZOID: contact zone identifier for visual analysis of protein-protein interactions
Source: BMC Bioinformatics. 2018 Apr 6;19:125. doi: 10.1186/s12859-018-2113-6 (PMC5889581; doi:10.1186/s12859-018-2113-6)
Supplement: Supplementary file 2 — Software build. Executable binary file of the software tool. (ZIP 118,784 kb) [file 12859_2018_2113_MOESM2_ESM.zip › cozoid/java/docs/beansbinding-1.2.1-doc.zip!/org/jdesktop/beansbinding/class-use/Property.html]

Uses of Class org.jdesktop.beansbinding.Property (Beans Binding)


---


|  |  |  |
| --- | --- | --- |
| **Overview** \| **Package** \| **Class** \| **Use** \| **Tree** \| **Deprecated** \| **Index** \| **Help** | |  |
| PREV   NEXT | **FRAMES**    **NO FRAMES**     **All Classes** |  |


---


## **Uses of Class org.jdesktop.beansbinding.Property**

| Packages that use Property | |
| --- | --- |
| **org.jdesktop.beansbinding** | Provides support for defining properties and creating bindings between sets of two properties. |
| **org.jdesktop.swingbinding** | Provides support for binding to complex Swing components, and documentation on the interesting Swing properties to bind to. |
| **org.jdesktop.swingbinding.impl** | Private implementation details for Swing binding. |

Uses of Property in org.jdesktop.beansbinding

| Subclasses of Property in org.jdesktop.beansbinding | |
| --- | --- |
| `class` | `BeanProperty<S,V>`             An implementation of `Property` that uses a simple dot-separated path syntax to address Java Beans properties of source objects. |
| `class` | `ELProperty<S,V>`             An implementation of `Property` that allows Java Beans properties of source objects to be addressed using a simple dot-separated path syntax within an EL expression. |
| `class` | `ObjectProperty<S>`             An immutable, read-only, `Property` implementation whose `getValue` method returns the source object that it is given. |
| `class` | `PropertyHelper<S,V>`             An abstract subclass of `Property` that helps with the management of `PropertyStateListeners` by implementing the methods for adding, removing, and getting listeners. |

| Methods in org.jdesktop.beansbinding that return Property | |
| --- | --- |
| `Property` | `PropertyStateEvent.getSourceProperty()`             Returns the `Property` whose state has changed. |
| `Property<SS,SV>` | `Binding.getSourceProperty()`             Returns the `Binding's` source property, which may not be `null`. |
| `Property<TS,TV>` | `Binding.getTargetProperty()`             Returns the `Binding's` target property, which may not be `null`. |

| Methods in org.jdesktop.beansbinding with parameters of type Property | |
| --- | --- |
| `static <S,V> ELProperty<S,V>` | `ELProperty.create(Property<S,?> baseProperty, java.lang.String expression)`             Creates an instance of `ELProperty` for the given base property and expression. |
| `static <S,V> BeanProperty<S,V>` | `BeanProperty.create(Property<S,?> baseProperty, java.lang.String path)`             Creates an instance of `BeanProperty` for the given base property and path. |
| `static <SS,SV,TS,TV> AutoBinding<SS,SV,TS,TV>` | `Bindings.createAutoBinding(AutoBinding.UpdateStrategy strategy, SS sourceObject, Property<SS,SV> sourceProperty, TS targetObject, Property<TS,TV> targetProperty)`             Creates an instance of `AutoBinding` that binds a property of a source object to a property of a target object. |
| `static <SS,SV,TS,TV> AutoBinding<SS,SV,TS,TV>` | `Bindings.createAutoBinding(AutoBinding.UpdateStrategy strategy, SS sourceObject, Property<SS,SV> sourceProperty, TS targetObject, Property<TS,TV> targetProperty)`             Creates an instance of `AutoBinding` that binds a property of a source object to a property of a target object. |
| `static <SS,SV,TS,TV> AutoBinding<SS,SV,TS,TV>` | `Bindings.createAutoBinding(AutoBinding.UpdateStrategy strategy, SS sourceObject, Property<SS,SV> sourceProperty, TS targetObject, Property<TS,TV> targetProperty, java.lang.String name)`             Creates a named instance of `AutoBinding` that binds a property of a source object to a property of a target object. |
| `static <SS,SV,TS,TV> AutoBinding<SS,SV,TS,TV>` | `Bindings.createAutoBinding(AutoBinding.UpdateStrategy strategy, SS sourceObject, Property<SS,SV> sourceProperty, TS targetObject, Property<TS,TV> targetProperty, java.lang.String name)`             Creates a named instance of `AutoBinding` that binds a property of a source object to a property of a target object. |
| `static <SS,TS,TV> AutoBinding<SS,SS,TS,TV>` | `Bindings.createAutoBinding(AutoBinding.UpdateStrategy strategy, SS sourceObject, TS targetObject, Property<TS,TV> targetProperty)`             Creates an instance of `AutoBinding` that binds a source object to a property of a target object. |
| `static <SS,TS,TV> AutoBinding<SS,SS,TS,TV>` | `Bindings.createAutoBinding(AutoBinding.UpdateStrategy strategy, SS sourceObject, TS targetObject, Property<TS,TV> targetProperty, java.lang.String name)`             Creates a named instance of `AutoBinding` that binds a source object to a property of a target object. |
| `protected  void` | `Binding.setSourceProperty(Property<SS,SV> sourceProperty)`             Sets the `Binding's` source property. |
| `protected  void` | `Binding.setTargetProperty(Property<TS,TV> targetProperty)`             Sets the `Binding's` target property. |

Constructors in org.jdesktop.beansbinding with parameters of type Property

`AutoBinding(AutoBinding.UpdateStrategy strategy,
SS sourceObject,
Property<SS,SV> sourceProperty,
TS targetObject,
Property<TS,TV> targetProperty,
java.lang.String name)`
  
          Create an instance of `AutoBinding` between two properties of two objects,
with the given update strategy.

`AutoBinding(AutoBinding.UpdateStrategy strategy,
SS sourceObject,
Property<SS,SV> sourceProperty,
TS targetObject,
Property<TS,TV> targetProperty,
java.lang.String name)`
  
          Create an instance of `AutoBinding` between two properties of two objects,
with the given update strategy.

`Binding(SS sourceObject,
Property<SS,SV> sourceProperty,
TS targetObject,
Property<TS,TV> targetProperty,
java.lang.String name)`
  
          Create an instance of `Binding` between two properties of two objects.

`Binding(SS sourceObject,
Property<SS,SV> sourceProperty,
TS targetObject,
Property<TS,TV> targetProperty,
java.lang.String name)`
  
          Create an instance of `Binding` between two properties of two objects.

`PropertyStateEvent(Property sourceProperty,
java.lang.Object sourceObject,
boolean valueChanged,
java.lang.Object oldValue,
java.lang.Object newValue,
boolean writeableChanged,
boolean isWriteable)`
  
          Creates an instance of `PropertyStateEvent` characterizing a
change in a `Property's` state for a particular source object.

Uses of Property in org.jdesktop.swingbinding

| Methods in org.jdesktop.swingbinding with parameters of type Property | |
| --- | --- |
| `JTableBinding.ColumnBinding` | `JTableBinding.addColumnBinding(int index, Property<E,?> columnProperty)`             Creates a `ColumnBinding` and inserts it at the given index into the list of `ColumnBindings` maintained by this `JTableBinding`. |
| `JTableBinding.ColumnBinding` | `JTableBinding.addColumnBinding(int index, Property<E,?> columnProperty, java.lang.String name)`             Creates a `ColumnBinding` and inserts it at the given index into the list of `ColumnBindings` maintained by this `JTableBinding`. |
| `JTableBinding.ColumnBinding` | `JTableBinding.addColumnBinding(Property<E,?> columnProperty)`             Creates a `ColumnBinding` and adds it to the end of the list of `ColumnBindings` maintained by this `JTableBinding`. |
| `JTableBinding.ColumnBinding` | `JTableBinding.addColumnBinding(Property<E,?> columnProperty, java.lang.String name)`             Creates a named `ColumnBinding` and adds it to the end of the list of `ColumnBindings` maintained by this `JTableBinding`. |
| `static <E,TS> JComboBoxBinding<E,java.util.List<E>,TS>` | `SwingBindings.createJComboBoxBinding(AutoBinding.UpdateStrategy strategy, java.util.List<E> sourceList, TS targetObject, Property<TS,? extends javax.swing.JComboBox> targetJComboBoxProperty)`             Creates a `JComboBoxBinding` from a direct reference to a `List` and an object and property that resolves to a `JComboBox`. |
| `static <E,TS> JComboBoxBinding<E,java.util.List<E>,TS>` | `SwingBindings.createJComboBoxBinding(AutoBinding.UpdateStrategy strategy, java.util.List<E> sourceList, TS targetObject, Property<TS,? extends javax.swing.JComboBox> targetJComboBoxProperty, java.lang.String name)`             Creates a named `JComboBoxBinding` from a direct reference to a `List` and an object and property that resolves to a `JComboBox`. |
| `static <E,SS> JComboBoxBinding<E,SS,javax.swing.JComboBox>` | `SwingBindings.createJComboBoxBinding(AutoBinding.UpdateStrategy strategy, SS sourceObject, Property<SS,java.util.List<E>> sourceListProperty, javax.swing.JComboBox targetJComboBox)`             Creates a `JComboBoxBinding` from an object and property that resolves to a `List` and a direct reference to a `JComboBox`. |
| `static <E,SS> JComboBoxBinding<E,SS,javax.swing.JComboBox>` | `SwingBindings.createJComboBoxBinding(AutoBinding.UpdateStrategy strategy, SS sourceObject, Property<SS,java.util.List<E>> sourceListProperty, javax.swing.JComboBox targetJComboBox, java.lang.String name)`             Creates a named `JComboBoxBinding` from an object and property that resolves to a `List` and a direct reference to a `JComboBox`. |
| `static <E,SS,TS> JComboBoxBinding<E,SS,TS>` | `SwingBindings.createJComboBoxBinding(AutoBinding.UpdateStrategy strategy, SS sourceObject, Property<SS,java.util.List<E>> sourceListProperty, TS targetObject, Property<TS,? extends javax.swing.JComboBox> targetJComboBoxProperty)`             Creates a `JComboBoxBinding` from an object and property that resolves to a `List` and an object and property that resolves to a `JComboBox`. |
| `static <E,SS,TS> JComboBoxBinding<E,SS,TS>` | `SwingBindings.createJComboBoxBinding(AutoBinding.UpdateStrategy strategy, SS sourceObject, Property<SS,java.util.List<E>> sourceListProperty, TS targetObject, Property<TS,? extends javax.swing.JComboBox> targetJComboBoxProperty)`             Creates a `JComboBoxBinding` from an object and property that resolves to a `List` and an object and property that resolves to a `JComboBox`. |
| `static <E,SS,TS> JComboBoxBinding<E,SS,TS>` | `SwingBindings.createJComboBoxBinding(AutoBinding.UpdateStrategy strategy, SS sourceObject, Property<SS,java.util.List<E>> sourceListProperty, TS targetObject, Property<TS,? extends javax.swing.JComboBox> targetJComboBoxProperty, java.lang.String name)`             Creates a named `JComboBoxBinding` from an object and property that resolves to a `List` and an object and property that resolves to a `JComboBox`. |
| `static <E,SS,TS> JComboBoxBinding<E,SS,TS>` | `SwingBindings.createJComboBoxBinding(AutoBinding.UpdateStrategy strategy, SS sourceObject, Property<SS,java.util.List<E>> sourceListProperty, TS targetObject, Property<TS,? extends javax.swing.JComboBox> targetJComboBoxProperty, java.lang.String name)`             Creates a named `JComboBoxBinding` from an object and property that resolves to a `List` and an object and property that resolves to a `JComboBox`. |
| `static <E,TS> JListBinding<E,java.util.List<E>,TS>` | `SwingBindings.createJListBinding(AutoBinding.UpdateStrategy strategy, java.util.List<E> sourceList, TS targetObject, Property<TS,? extends javax.swing.JList> targetJListProperty)`             Creates a `JListBinding` from a direct reference to a `List` and an object and property that resolves to a `JList`. |
| `static <E,TS> JListBinding<E,java.util.List<E>,TS>` | `SwingBindings.createJListBinding(AutoBinding.UpdateStrategy strategy, java.util.List<E> sourceList, TS targetObject, Property<TS,? extends javax.swing.JList> targetJListProperty, java.lang.String name)`             Creates a named `JListBinding` from a direct reference to a `List` and an object and property that resolves to a `JList`. |
| `static <E,SS> JListBinding<E,SS,javax.swing.JList>` | `SwingBindings.createJListBinding(AutoBinding.UpdateStrategy strategy, SS sourceObject, Property<SS,java.util.List<E>> sourceListProperty, javax.swing.JList targetJList)`             Creates a `JListBinding` from an object and property that resolves to a `List` and a direct reference to a `JList`. |
| `static <E,SS> JListBinding<E,SS,javax.swing.JList>` | `SwingBindings.createJListBinding(AutoBinding.UpdateStrategy strategy, SS sourceObject, Property<SS,java.util.List<E>> sourceListProperty, javax.swing.JList targetJList, java.lang.String name)`             Creates a named `JListBinding` from an object and property that resolves to a `List` and a direct reference to a `JList`. |
| `static <E,SS,TS> JListBinding<E,SS,TS>` | `SwingBindings.createJListBinding(AutoBinding.UpdateStrategy strategy, SS sourceObject, Property<SS,java.util.List<E>> sourceListProperty, TS targetObject, Property<TS,? extends javax.swing.JList> targetJListProperty)`             Creates a `JListBinding` from an object and property that resolves to a `List` and an object and property that resolves to a `JList`. |
| `static <E,SS,TS> JListBinding<E,SS,TS>` | `SwingBindings.createJListBinding(AutoBinding.UpdateStrategy strategy, SS sourceObject, Property<SS,java.util.List<E>> sourceListProperty, TS targetObject, Property<TS,? extends javax.swing.JList> targetJListProperty)`             Creates a `JListBinding` from an object and property that resolves to a `List` and an object and property that resolves to a `JList`. |
| `static <E,SS,TS> JListBinding<E,SS,TS>` | `SwingBindings.createJListBinding(AutoBinding.UpdateStrategy strategy, SS sourceObject, Property<SS,java.util.List<E>> sourceListProperty, TS targetObject, Property<TS,? extends javax.swing.JList> targetJListProperty, java.lang.String name)`             Creates a named `JListBinding` from an object and property that resolves to a `List` and an object and property that resolves to a `JList`. |
| `static <E,SS,TS> JListBinding<E,SS,TS>` | `SwingBindings.createJListBinding(AutoBinding.UpdateStrategy strategy, SS sourceObject, Property<SS,java.util.List<E>> sourceListProperty, TS targetObject, Property<TS,? extends javax.swing.JList> targetJListProperty, java.lang.String name)`             Creates a named `JListBinding` from an object and property that resolves to a `List` and an object and property that resolves to a `JList`. |
| `static <E,TS> JTableBinding<E,java.util.List<E>,TS>` | `SwingBindings.createJTableBinding(AutoBinding.UpdateStrategy strategy, java.util.List<E> sourceList, TS targetObject, Property<TS,? extends javax.swing.JTable> targetJTableProperty)`             Creates a `JTableBinding` from a direct reference to a `List` and an object and property that resolves to a `JTable`. |
| `static <E,TS> JTableBinding<E,java.util.List<E>,TS>` | `SwingBindings.createJTableBinding(AutoBinding.UpdateStrategy strategy, java.util.List<E> sourceList, TS targetObject, Property<TS,? extends javax.swing.JTable> targetJTableProperty, java.lang.String name)`             Creates a named `JTableBinding` from a direct reference to a `List` and an object and property that resolves to a `JTable`. |
| `static <E,SS> JTableBinding<E,SS,javax.swing.JTable>` | `SwingBindings.createJTableBinding(AutoBinding.UpdateStrategy strategy, SS sourceObject, Property<SS,java.util.List<E>> sourceListProperty, javax.swing.JTable targetJTable)`             Creates a `JTableBinding` from an object and property that resolves to a `List` and a direct reference to a `JTable`. |
| `static <E,SS> JTableBinding<E,SS,javax.swing.JTable>` | `SwingBindings.createJTableBinding(AutoBinding.UpdateStrategy strategy, SS sourceObject, Property<SS,java.util.List<E>> sourceListProperty, javax.swing.JTable targetJTable, java.lang.String name)`             Creates a named `JTableBinding` from an object and property that resolves to a `List` and a direct reference to a `JTable`. |
| `static <E,SS,TS> JTableBinding<E,SS,TS>` | `SwingBindings.createJTableBinding(AutoBinding.UpdateStrategy strategy, SS sourceObject, Property<SS,java.util.List<E>> sourceListProperty, TS targetObject, Property<TS,? extends javax.swing.JTable> targetJTableProperty)`             Creates a `JTableBinding` from an object and property that resolves to a `List` and an object and property that resolves to a `JTable`. |
| `static <E,SS,TS> JTableBinding<E,SS,TS>` | `SwingBindings.createJTableBinding(AutoBinding.UpdateStrategy strategy, SS sourceObject, Property<SS,java.util.List<E>> sourceListProperty, TS targetObject, Property<TS,? extends javax.swing.JTable> targetJTableProperty)`             Creates a `JTableBinding` from an object and property that resolves to a `List` and an object and property that resolves to a `JTable`. |
| `static <E,SS,TS> JTableBinding<E,SS,TS>` | `SwingBindings.createJTableBinding(AutoBinding.UpdateStrategy strategy, SS sourceObject, Property<SS,java.util.List<E>> sourceListProperty, TS targetObject, Property<TS,? extends javax.swing.JTable> targetJTableProperty, java.lang.String name)`             Creates a named `JTableBinding` from an object and property that resolves to a `List` and an object and property that resolves to a `JTable`. |
| `static <E,SS,TS> JTableBinding<E,SS,TS>` | `SwingBindings.createJTableBinding(AutoBinding.UpdateStrategy strategy, SS sourceObject, Property<SS,java.util.List<E>> sourceListProperty, TS targetObject, Property<TS,? extends javax.swing.JTable> targetJTableProperty, java.lang.String name)`             Creates a named `JTableBinding` from an object and property that resolves to a `List` and an object and property that resolves to a `JTable`. |
| `JListBinding.DetailBinding` | `JListBinding.setDetailBinding(Property<E,?> detailProperty)`             Creates a `DetailBinding` and sets it as the `DetailBinding` for this `JListBinding`. |
| `JListBinding.DetailBinding` | `JListBinding.setDetailBinding(Property<E,?> detailProperty, java.lang.String name)`             Creates a named `DetailBinding` and sets it as the `DetailBinding` for this `JListBinding`. |

Constructors in org.jdesktop.swingbinding with parameters of type Property

`JComboBoxBinding(AutoBinding.UpdateStrategy strategy,
SS sourceObject,
Property<SS,java.util.List<E>> sourceListProperty,
TS targetObject,
Property<TS,? extends javax.swing.JComboBox> targetJComboBoxProperty,
java.lang.String name)`
  
          Constructs an instance of `JComboBoxBinding`.

`JComboBoxBinding(AutoBinding.UpdateStrategy strategy,
SS sourceObject,
Property<SS,java.util.List<E>> sourceListProperty,
TS targetObject,
Property<TS,? extends javax.swing.JComboBox> targetJComboBoxProperty,
java.lang.String name)`
  
          Constructs an instance of `JComboBoxBinding`.

`JListBinding(AutoBinding.UpdateStrategy strategy,
SS sourceObject,
Property<SS,java.util.List<E>> sourceListProperty,
TS targetObject,
Property<TS,? extends javax.swing.JList> targetJListProperty,
java.lang.String name)`
  
          Constructs an instance of `JListBinding`.

`JListBinding(AutoBinding.UpdateStrategy strategy,
SS sourceObject,
Property<SS,java.util.List<E>> sourceListProperty,
TS targetObject,
Property<TS,? extends javax.swing.JList> targetJListProperty,
java.lang.String name)`
  
          Constructs an instance of `JListBinding`.

`JTableBinding(AutoBinding.UpdateStrategy strategy,
SS sourceObject,
Property<SS,java.util.List<E>> sourceListProperty,
TS targetObject,
Property<TS,? extends javax.swing.JTable> targetJTableProperty,
java.lang.String name)`
  
          Constructs an instance of `JTableBinding`.

`JTableBinding(AutoBinding.UpdateStrategy strategy,
SS sourceObject,
Property<SS,java.util.List<E>> sourceListProperty,
TS targetObject,
Property<TS,? extends javax.swing.JTable> targetJTableProperty,
java.lang.String name)`
  
          Constructs an instance of `JTableBinding`.

Uses of Property in org.jdesktop.swingbinding.impl

Constructors in org.jdesktop.swingbinding.impl with parameters of type Property

`AbstractColumnBinding(int column,
Property columnSource,
Property columnTarget,
java.lang.String name)`

`AbstractColumnBinding(int column,
Property columnSource,
Property columnTarget,
java.lang.String name)`

---


|  |  |  |
| --- | --- | --- |
| **Overview** \| **Package** \| **Class** \| **Use** \| **Tree** \| **Deprecated** \| **Index** \| **Help** | |  |
| PREV   NEXT | **FRAMES**    **NO FRAMES**     **All Classes** |  |


---
